# Halogen-bonded network of trinuclear copper(II) 4-iodo­pyrazolate complexes formed by mutual breakdown of chloro­form and nanojars

**DOI:** 10.1107/S205698901601536X

**Published:** 2016-10-04

**Authors:** Stuart A. Surmann, Gellert Mezei

**Affiliations:** a Department of Chemistry, Western Michigan University, Kalamazoo, MI 49006

**Keywords:** crystal structure, copper pyrazolate, trinuclear complex, nanojar, halogen bonding

## Abstract

Crystals of bis­(tetra­butyl­ammonium) di-μ_3_-chlorido-­tris­(μ_2_-4-iodo­pyrazolato-κ^2^ *N*:*N*′)tris­[chlorido­cuprate(II)] 1,4-dioxane hemisolvate, (C_16_H_36_N)_2_[Cu_3_(C_3_H_2_IN_2_)_3_Cl_5_]·0.5C_4_H_8_O or (Bu_4_N)_2_[Cu^II^_3_(*μ*_3_-Cl)_2_(*μ*-4-I-pz)_3_Cl_3_]·0.5C_4_H_8_O, were obtained by evaporating a solution of (Bu_4_N)_2_[{Cu^II^(*μ*-OH)(*μ*-4-I-pz)}_*n*_CO_3_] (*n* = 27–31) nanojars in chloro­form/1,4-dioxane. The decomposition of chloro­form in the presence of oxygen and moisture provides HCl, which leads to the breakdown of nanojars to the title trinuclear copper(II) pyrazolate complex, and possibly Cu^II^ ions and free 4-iodo­pyrazole. Cu^II^ ions, in turn, act as catalyst for the accelerated decomposition of chloro­form, ultimately leading to the complete breakdown of nanojars. The crystal structure presented here provides the first structural description of a trinuclear copper(II) pyrazolate complex with iodine-substituted pyrazoles. In contrast to related trinuclear complexes based on differently substituted 4-*R*-pyrazoles (*R* = H, Cl, Br, Me), the [Cu_3_(*μ*-4-I-pz)_3_Cl_3_] core in the title complex is nearly planar. This difference is likely a result of the presence of the iodine substituent, which provides a unique, novel feature in copper pyrazolate chemistry. Thus, the iodine atoms form halogen bonds with the terminal chlorido ligands of the surrounding complexes [mean length of I⋯Cl contacts = 3.48 (1) Å], leading to an extended two-dimensional, halogen-bonded network along (-110). The cavities within this framework are filled by centrosymmetric 1,4-dioxane solvent mol­ecules, which create further bridges *via* C—H⋯Cl hydrogen bonds with terminal chlorido ligands of the trinuclear complex not involved in halogen bonding.

## Abstract 2

Acidity created by the decomposition of chloro­form solvent leads to breakdown of (Bu_4_N)_2_[{Cu^II^(*μ*-OH)(*μ*-4-I-pz)}_*n*_CO_3_] (*n* = 27–31) nanojars in a chloro­form/1,4-dioxane solution to the trinuclear complex (Bu_4_N)_2_[Cu_3_(*μ*_3_-Cl)_2_(*μ*-4-I-pz)_3_Cl_3_]·0.5dioxane, which forms extended sheets based on C—I⋯Cl—Cu halogen bonding and C—H⋯Cl—Cu hydrogen bonding.

## Chemical context   

Nanojars, supra­molecular coordination complexes of the formula [{Cu(*μ*-OH)(*μ*-pz)}_*n*_anion] (pz = pyrazolate anion; *n* = 27–36), have emerged as a new class of anion encapsulation agents of unparalleled efficiency, which allow the extraction of anions with large hydration energies, such as phosphate, carbonate and sulfate, from water into organic solvents (Mezei, Baran *et al.*, 2004 ▸; Fernando *et al.*, 2012 ▸; Mezei, 2015 ▸; Ahmed, Szymczyna *et al.*, 2016 ▸; Ahmed, Calco & Mezei, 2016 ▸; Ahmed & Mezei, 2016 ▸; Ahmed, Hartman & Mezei, 2016 ▸). Trinuclear copper pyrazolate complexes have been identified as key inter­mediates in the self-assembly mechanism of nanojars from copper(II) nitrate, pyrazole and NaOH (1:1:2 molar ratio) in the presence of carbonate (Ahmed & Mezei, 2016 ▸). The trinuclear inter­mediate can be isolated if the amount of available base is reduced (copper:pyrazole:base molar ratio 3:3:4), and can subsequently be converted to nanojars by adding an additional amount of base to reach a 1:1:2 molar ratio. Moreover, nanojars can be broken down to the trinuclear complex by acids, which easily proton­ate the OH groups of the nanojar. As a consequence, nanojars and the trinuclear pyrazolate complex are in a pH-dependent equilibrium. The sensitivity of nanojars to even very weak acids is further demonstrated by the fact that a weak base, such as Et_3_N, is unable to convert the trinuclear complex to nanojars in solution (*e.g*., DMF, THF), despite its ability to provide the hydroxide ions needed by the nanojar, in the presence of moisture (Et_3_N + H_2_O 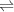 Et_3_NH^+^ + HO^−^). This is due to the acidity of the conjugate acid, the tri­ethyl­ammonium cation (p*K*_a_ = 10.75 in H_2_O), which would form in the process (Mezei, 2016 ▸). Nevertheless, nanojars can be obtained using Et_3_N if the solution is diluted with excess water, which leads to the precipitation of hydro­phobic nanojars (Fernando *et al.*, 2012 ▸).
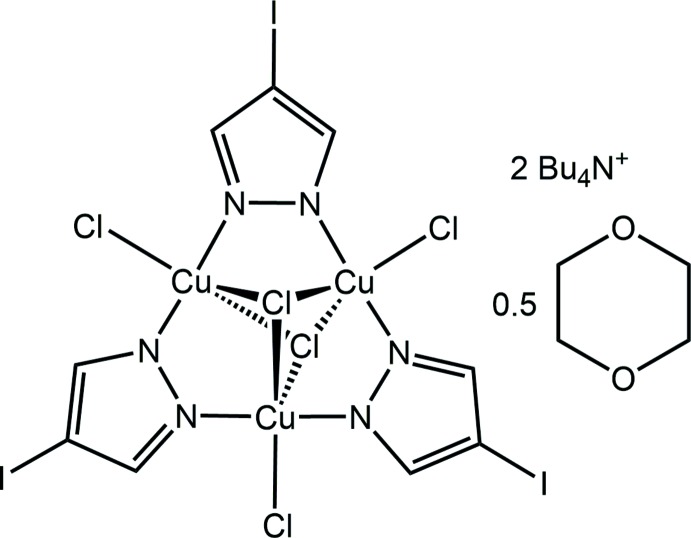


New evidence supporting the vulnerability of nanojars to acids emerges from an unexpected source. An attempt to grow single crystals from a solution of (Bu_4_N)_2_[{Cu(*μ*-OH)(*μ*-4-I-pz)}_*n*_CO_3_] (*n* = 27–31) (Ahmed, Calco *et al.*, 2016 ▸) in chloro­form/1,4-dioxane provided, instead of the expected nanojars, crystals of (Bu_4_N)_2_[Cu_3_(*μ*_3_-Cl)_2_(*μ*-4-I-pz)_3_Cl_3_]·0.5dioxane (Mezei & Raptis, 2004 ▸), accompanied by a color change of the solution from blue to green. The chloride ions originating from CHCl_3_ is not surprising, as chloro­form has long been known to slowly decompose in the presence of air and moisture producing HCl and phosgene (CHCl_3_ + ½O_2_ → COCl_2_ + HCl) (Baskerville & Hamor, 1912 ▸). The latter can hydrolyze to provide further amounts of HCl, and CO_2_ (COCl_2_ + H_2_O → 2HCl + CO_2_). What is surprising though is the large amount of chloride formed in a relatively short period of time (*ca* 48 chloride ions per nanojar). Chloro­form preserved with ethanol (0.5–1%), such as the one used here for crystal growing, is much more stable than the pure form and it does not decompose at a significant rate. This points to a decomposition catalyzed by the dissolved nanojars, possibly aided by light. A search of the literature shows that various classes of compounds have been found to catalyze the photodecomposition of chloro­form (Semeluk & Unger, 1963 ▸; Peña & Hoggard, 2010 ▸; Muñoz *et al.*, 2008 ▸; Peña *et al.*, 2014 ▸; Peña *et al.*, 2009 ▸), including simple copper(II) complexes (Harvey & Hoggard, 2014 ▸). A balanced equation of the reaction between nanojars of different sizes and HCl, producing the title trinuclear complex, is given below:

3[{Cu(*μ*-OH)(*μ*-4-Ipz)_*n*_CO_3_]^2–^ + 5*n*HCl → *n*[Cu_3_(*μ*_3_-Cl)_2_(*μ*-4-Ipz)_3_Cl_3_]^2–^ + (2*n* − 6) H_3_O^+^ + (*n* + 9) H_2_O + 3CO_2_ (*n* = 27–31).

## Structural commentary   

**Figure 1:**
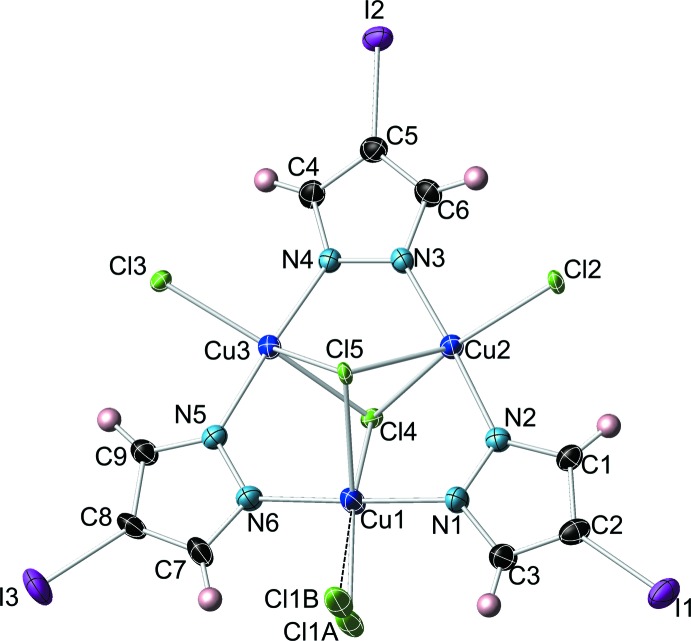
Displacement ellipsoid plot (50% probability level) of the title trinuclear copper pyrazolate complex anion, showing the atom-labeling scheme (counter-ions and solvent mol­ecule omitted).

The title compound contains a nearly planar Cu_3_(*μ*-4-I-pz)_3_ core (Fig. 1 ▸): the best-fit planes of the three 4-iodo­pyrazolate units form dihedral angles of 2.1 (2), 2.0 (1) and 6.5 (1)°, respectively, with the Cu_3_-plane. Each Cu atom has a distorted trigonal–bipyramidal coordination geometry and is bound to a terminal Cl atom (one Cl atom disordered over two positions, 60/40 occupancy) at an average Cu—Cl distance of 2.32 (3) Å. The Cu_3_ unit is additionally capped by two Cl atoms, one on each side of the complex, at distances of 1.683 (1) and 1.799 (1) Å from the Cu_3_-plane, respectively [average Cu—Cl distances = 2.58 (7) and 2.66 (9) Å]. The two capping Cl atoms impart an overall 2– charge to the complex, which is balanced by two tetra­butyl­ammonium counter-ions. Other bond lengths and angles within the Cu_3_(*μ_3_*-Cl)_2_(*μ*-4-I-pz)_3_Cl_3_ complex are similar to the ones found in related complexes (Angaridis *et al.*, 2002 ▸; Mezei & Raptis, 2004 ▸; Mezei *et al.*, 2006 ▸): Cu—N bond lengths average 1.936 (10) Å, N—Cu—N angles average 173 (3)°, Cl—Cu—Cl angles average 125 (9) and 152 (9)°, respectively, and intra­molecular Cu⋯Cu distances are 3.378 (1), 3.419 (1) and 3.390 (1) Å.

## Supra­molecular features   

**Figure 2:**
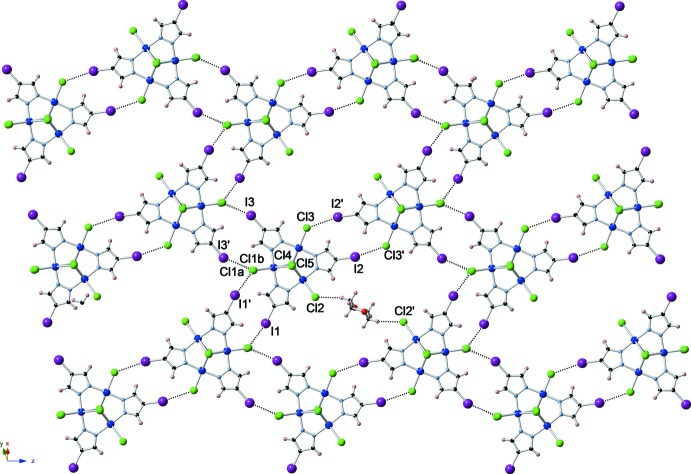
Two-dimensional sheet [along (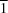
10)] formed by inter­molecular iodine–chlorine halogen bonding (only one dioxane solvent mol­ecule and no counter-ions are shown). Halogen bonds and C—H⋯Cl hydrogen bonds are indicated by dotted lines.

The inter­molecular distances between iodine substituents of the pyrazole units and the terminal chlorine atoms of adjacent complexes are less than the sum of the van der Waals radii (Bondi, 1964 ▸) of iodine and chlorine atoms (3.73 Å). Thus, a halogen-bonded (Cavallo *et al.*, 2016 ▸; Gilday *et al.*, 2015 ▸) sheet based on C—I⋯Cl—Cu inter­actions (Fig. 2 ▸) is generated parallel to the (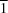
10) plane (and *c* axis); I⋯Cl distances and C–I⋯Cl angles are shown in Table 1 ▸. Bifurcated halogen bonds are noted between Cl1*A*/Cl1*B* and I1′ and I3′. The formation of the extended halogen-bonded network might account for the near-planarity of the title complex, as opposed to related complexes with unsubstituted or differently substituted 4-*R*-pyrazoles (*R* = H, Cl, Br, Me; Angaridis *et al.*, 2002 ▸; Mezei & Raptis, 2004 ▸), which do not form inter­molecular halogen bonds and are severely distorted from planarity. Additionally, the dioxane solvent mol­ecule, which is located around an inversion center, forms C—H⋯Cl hydrogen bonds with terminal chlorido ligands of the trinuclear complex [C43⋯Cl2: 3.751 (10); H43*B*⋯Cl2: 2.83; C43—H43*B*: 0.97 Å; C43—H43b⋯Cl2: 160 (5)°], creating further bridges within the two-dimensional framework.

## Database survey   

A search of the Cambridge Structural Database (Groom *et al.*, 2016 ▸) reveals only seven metal complexes that contain a 4-iodo­pyrazole moiety, either in its neutral, monodentate form (Guzei & Winter, 1997 ▸; Govor *et al.*, 2012 ▸; Song *et al.*, 2013 ▸; da Silva *et al.*, 2015 ▸), or in its deprotonated, bidentate form (Heeg *et al.*, 2010 ▸; Song *et al.*, 2013 ▸). Of these, only one is a Cu^II^ complex (Song *et al.*, 2013 ▸). Hence, the crystal structure presented here offers the first solid-state structural description of a trinuclear copper(II) pyrazolate complex bearing 4-iodo­pyrazolate ligands.

## Synthesis and crystallization   

The synthesis of (Bu_4_N)_2_[{Cu(μ-OH)(μ-4-I-pz)}_*n*_CO_3_] (*n* = 27–31) was described earlier (Ahmed Calco & Mezei, 2016 ▸). Green plate-like crystals of the title compound were obtained by slow evaporation of a chloro­form/1,4-dioxane (1 mL each) solution of (Bu_4_N)_2_[{Cu(μ-OH)(μ-4-I-pz)}_*n*_CO_3_] (20 mg).

## Refinement   

Crystal data, data collection and structure refinement details are summarized in Table 2 ▸. C—H hydrogen atoms were placed in idealized positions and refined using a riding model. One of the three terminal Cl-atoms is disordered over two positions (60/40). Two terminal CH_2_CH_3_ groups of one tetra­butyl­ammonium counter-ion and another CH_2_CH_3_ group of the other counter-ion are disordered over two positions (60/40); C—H bond-length restraints were used for the disordered C atoms. Residual electron density of 3.52 eÅ^−3^ is found at 0.83 Å from heavy atom I3, due to Fourier truncation ripples.

## Supplementary Material

Crystal structure: contains datablock(s) I. DOI: 10.1107/S205698901601536X/gk2666sup1.cif

Structure factors: contains datablock(s) I. DOI: 10.1107/S205698901601536X/gk2666Isup2.hkl

Supporting information file. DOI: 10.1107/S205698901601536X/gk2666Isup3.cdx

CCDC reference: 1507382

Additional supporting information: crystallographic information; 3D view; checkCIF report

## Figures and Tables

**Table 1:** Halogen-bond geometry (Å, °)

*D*—*X*⋯*Y*	*X*⋯*Y*	*D*—*X*⋯*Y*
C2—I1⋯Cl1*A* ^i^	3.516 (4)	152.0 (2)
C2—I1⋯Cl1*B* ^i^	3.362 (5)	164.3 (2)
C5—I2⋯Cl3^iii^	3.569 (1)	165.2 (2)
C8—I3⋯Cl1*A* ^iv^	3.438 (4)	154.4 (2)
C8—I3⋯Cl1*B* ^iv^	3.486 (5)	154.2 (2)

Symmetry codes: (i) −*x* + 2, −*y*, −*z*; (ii) −*x* + 1, −*y* − 1, −*z* + 1; (iii) −*x* + 1, −*y* − 1, −*z* + 1; (iv) −*x* + 1, −*y* − 1, −*z*.

**Table 2:** Experimental details

Crystal data	
Chemical formula	(C_16_H_36_N)_2_[Cu_3_(C_3_H_2_IN_2_)_3_Cl_5_]·0.5C_4_H_8_O
*M*_r_	1475.73
Crystal system, space group	Triclinic, *P* 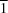
Temperature (K)	100
*a*, *b*, *c* (Å)	11.3604 (2), 11.5688 (2), 23.2200 (3)
α, β, γ (°)	103.707 (1), 90.409 (1), 93.654 (1)
*V* (Å^3^)	2958.00 (8)
*Z*	2
Radiation type	Mo *K*α
μ (mm^−1^)	2.90
Crystal size (mm)	0.65 × 0.43 × 0.03

Data collection	
Diffractometer	Bruker APEXII CCD
Absorption correction	Multi-scan (*SADABS*; Bruker, 2014 ▸)
*T*_min_, *T*_max_	0.486, 0.746
No. of measured, independent and observed [*I* > 2σ(*I*)] reflections	136857, 14685, 11845
*R*_int_	0.056
(sin θ/λ)_max_ (Å^−1^)	0.668

Refinement	
*R*[*F* ^2^ > 2σ(*F* ^2^)], *wR*(*F* ^2^), *S*	0.054, 0.153, 1.02
No. of reflections	14685
No. of parameters	642
No. of restraints	12
H-atom treatment	H-atom parameters constrained
Δρ_max_, Δρ_min_ (e Å^−3^)	3.54, −2.89

Computer programs: *APEX2* and *SAINT* (Bruker, 2014 ▸), *SHELXS97* (Sheldrick, 2008 ▸), *SHELXL2014/6* (Sheldrick, 2015 ▸) and *CrystalMaker* (Palmer, 2014 ▸).

## supplementary crystallographic information

### Crystal data

**Table:**

(C_16_H_36_N)_2_[Cu_3_Cl_5_(C_3_H_2_IN_2_)_3_Cl_5_]·0.5C_4_H_8_O	*Z* = 2
*M**_r_* = 1475.73	*F*(000) = 1470
Triclinic, *P*1	*D*_x_ = 1.657 Mg m^−^^3^
*a* = 11.3604 (2) Å	Mo *K*α radiation, λ = 0.71073 Å
*b* = 11.5688 (2) Å	Cell parameters from 9763 reflections
*c* = 23.2200 (3) Å	θ = 2.2–28.1°
α = 103.707 (1)°	µ = 2.90 mm^−^^1^
β = 90.409 (1)°	*T* = 100 K
γ = 93.654 (1)°	Plate, green
*V* = 2958.00 (8) Å^3^	0.65 × 0.43 × 0.03 mm

### Data collection

**Table:**

Bruker APEXII CCD diffractometer	11845 reflections with *I* > 2σ(*I*)
φ and ω scans	*R*_int_ = 0.056
Absorption correction: multi-scan (SADABS; Bruker, 2014)	θ_max_ = 28.3°, θ_min_ = 0.9°
*T*_min_ = 0.486, *T*_max_ = 0.746	*h* = −15→15
136857 measured reflections	*k* = −15→15
14685 independent reflections	*l* = −30→30

### Refinement

**Table:**

Refinement on *F*^2^	12 restraints
Least-squares matrix: full	Hydrogen site location: inferred from neighbouring sites
*R*[*F*^2^ > 2σ(*F*^2^)] = 0.054	H-atom parameters constrained
*wR*(*F*^2^) = 0.153	*w* = 1/[σ^2^(*F*_o_^2^) + (0.0646*P*)^2^ + 28.784*P*] where *P* = (*F*_o_^2^ + 2*F*_c_^2^)/3
*S* = 1.02	(Δ/σ)_max_ = 0.001
14685 reflections	Δρ_max_ = 3.54 e Å^−^^3^
642 parameters	Δρ_min_ = −2.89 e Å^−^^3^

### Special details

**Table:**

Geometry. All esds (except the esd in the dihedral angle between two l.s. planes) are estimated using the full covariance matrix. The cell esds are taken into account individually in the estimation of esds in distances, angles and torsion angles; correlations between esds in cell parameters are only used when they are defined by crystal symmetry. An approximate (isotropic) treatment of cell esds is used for estimating esds involving l.s. planes.

### Fractional atomic coordinates and isotropic or equivalent isotropic displacement parameters (Å^2^)

**Table:**

	*x*	*y*	*z*	*U*_iso_*/*U*_eq_	Occ. (<1)
I1	1.03331 (4)	1.16081 (4)	0.94854 (2)	0.03491 (11)	
I2	0.60033 (4)	0.67453 (3)	0.48116 (2)	0.02938 (10)	
I3	0.33478 (5)	0.37443 (5)	0.92175 (2)	0.05065 (15)	
Cu1	0.68348 (7)	0.74045 (8)	0.86260 (3)	0.03445 (19)	
Cu2	0.76595 (6)	0.82666 (6)	0.73883 (3)	0.02209 (14)	
Cu3	0.54352 (6)	0.60997 (6)	0.73172 (3)	0.01939 (14)	
Cl1A	0.7088 (4)	0.7605 (4)	0.96245 (14)	0.0517 (10)	0.6
Cl1B	0.7610 (5)	0.7019 (5)	0.9490 (2)	0.0456 (13)	0.4
Cl2	0.91814 (10)	0.91245 (10)	0.69157 (5)	0.0170 (2)	
Cl3	0.43631 (10)	0.44226 (10)	0.67503 (5)	0.0154 (2)	
Cl4	0.55813 (9)	0.82369 (10)	0.78789 (5)	0.0150 (2)	
Cl5	0.77907 (9)	0.62234 (9)	0.76421 (4)	0.01119 (19)	
O1	0.9089 (5)	0.0637 (5)	0.5297 (2)	0.0456 (12)	
N1	0.7933 (4)	0.8756 (5)	0.8653 (2)	0.0274 (10)	
N2	0.8247 (4)	0.9129 (4)	0.81648 (19)	0.0208 (9)	
N3	0.6824 (4)	0.7512 (4)	0.66521 (19)	0.0213 (9)	
N4	0.5915 (4)	0.6671 (4)	0.66256 (19)	0.0207 (9)	
N5	0.5023 (4)	0.5613 (4)	0.80432 (19)	0.0223 (9)	
N6	0.5610 (4)	0.6148 (5)	0.8565 (2)	0.0269 (10)	
N7	0.1835 (4)	0.7648 (5)	0.8299 (3)	0.0336 (12)	
N8	0.7491 (5)	0.2456 (5)	0.6678 (3)	0.0338 (12)	
C1	0.9002 (5)	1.0111 (5)	0.8331 (2)	0.0219 (10)	
H1	0.9350	1.0540	0.8078	0.026*	
C2	0.9171 (5)	1.0369 (5)	0.8945 (2)	0.0232 (11)	
C3	0.8487 (5)	0.9500 (6)	0.9130 (3)	0.0295 (13)	
H3	0.8418	0.9436	0.9520	0.035*	
C4	0.5500 (5)	0.6341 (5)	0.6066 (2)	0.0227 (11)	
H4	0.4874	0.5782	0.5929	0.027*	
C5	0.6150 (5)	0.6965 (5)	0.5720 (2)	0.0233 (11)	
C6	0.6974 (5)	0.7683 (5)	0.6107 (2)	0.0232 (11)	
H6	0.7545	0.8206	0.6004	0.028*	
C7	0.5171 (5)	0.5648 (6)	0.8991 (3)	0.0294 (12)	
H7	0.5424	0.5846	0.9386	0.035*	
C8	0.4282 (6)	0.4787 (6)	0.8748 (3)	0.0314 (13)	
C9	0.4229 (5)	0.4791 (5)	0.8146 (2)	0.0260 (11)	
H9	0.3721	0.4299	0.7863	0.031*	
C10	0.0713 (6)	0.7144 (9)	0.8526 (4)	0.056 (2)	
H10A	0.0536	0.7664	0.8905	0.067*	
H10B	0.0069	0.7160	0.8252	0.067*	
C11	0.0750 (8)	0.5893 (13)	0.8606 (7)	0.104 (5)	
H11A	0.1434	0.5818	0.8844	0.125*	0.6
H11B	0.0769	0.5321	0.8226	0.125*	0.6
H11C	0.1322	0.5965	0.8927	0.125*	0.4
H11D	0.1128	0.5470	0.8252	0.125*	0.4
C12A	−0.0422 (11)	0.5702 (14)	0.8934 (7)	0.047 (3)	0.6
H12A	−0.1102	0.5837	0.8710	0.056*	0.6
H12B	−0.0415	0.6233	0.9326	0.056*	0.6
C13A	−0.0432 (18)	0.4346 (17)	0.8972 (12)	0.112 (10)	0.6
H13A	−0.0392	0.3843	0.8580	0.168*	0.6
H13B	−0.1146	0.4136	0.9153	0.168*	0.6
H13C	0.0235	0.4241	0.9206	0.168*	0.6
C12B	−0.020 (3)	0.500 (3)	0.8721 (10)	0.077 (9)	0.4
H12C	−0.0074	0.4199	0.8491	0.093*	0.4
H12D	−0.0977	0.5209	0.8630	0.093*	0.4
C13B	−0.002 (3)	0.510 (3)	0.9386 (10)	0.087 (9)	0.4
H13D	0.0684	0.4729	0.9453	0.131*	0.4
H13E	−0.0687	0.4705	0.9530	0.131*	0.4
H13F	0.0047	0.5924	0.9593	0.131*	0.4
C14	0.2866 (6)	0.7694 (7)	0.8727 (3)	0.0363 (14)	
H14A	0.3572	0.7979	0.8556	0.044*	
H14B	0.2980	0.6886	0.8758	0.044*	
C15	0.2755 (7)	0.8467 (9)	0.9350 (3)	0.056 (2)	
H15A	0.2121	0.8136	0.9552	0.067*	0.6
H15B	0.2575	0.9267	0.9332	0.067*	0.6
H15C	0.1961	0.8357	0.9489	0.067*	0.4
H15D	0.2903	0.9301	0.9348	0.067*	0.4
C16A	0.394 (2)	0.851 (4)	0.9692 (11)	0.16 (2)	0.6
H16A	0.4103	0.7700	0.9698	0.192*	0.6
H16B	0.4560	0.8815	0.9473	0.192*	0.6
C17A	0.400 (2)	0.926 (3)	1.0325 (9)	0.172 (18)	0.6
H17A	0.3964	1.0084	1.0323	0.258*	0.6
H17B	0.4719	0.9143	1.0513	0.258*	0.6
H17C	0.3340	0.9018	1.0538	0.258*	0.6
C16B	0.367 (2)	0.811 (3)	0.9773 (13)	0.058 (8)	0.4
H16C	0.4381	0.7876	0.9565	0.069*	0.4
H16D	0.3867	0.8774	1.0109	0.069*	0.4
C17B	0.306 (3)	0.705 (3)	0.9983 (12)	0.086 (9)	0.4
H17D	0.2298	0.7260	1.0136	0.130*	0.4
H17E	0.3536	0.6868	1.0288	0.130*	0.4
H17F	0.2965	0.6367	0.9654	0.130*	0.4
C18	0.2168 (6)	0.6875 (6)	0.7706 (3)	0.0341 (13)	
H18A	0.2361	0.6105	0.7763	0.041*	
H18B	0.2872	0.7241	0.7571	0.041*	
C19	0.1213 (7)	0.6679 (7)	0.7222 (4)	0.0442 (17)	
H19A	0.0830	0.7414	0.7245	0.053*	
H19B	0.0622	0.6076	0.7279	0.053*	
C20	0.1771 (9)	0.6273 (9)	0.6606 (4)	0.067 (3)	
H20A	0.1148	0.5955	0.6312	0.080*	
H20B	0.2281	0.5636	0.6615	0.080*	
C21	0.2499 (11)	0.7297 (13)	0.6413 (5)	0.088 (4)	
H21A	0.2812	0.6998	0.6027	0.133*	
H21B	0.3137	0.7596	0.6693	0.133*	
H21C	0.1999	0.7928	0.6400	0.133*	
C22	0.1610 (6)	0.8890 (6)	0.8236 (3)	0.0389 (15)	
H22A	0.1364	0.9351	0.8616	0.047*	
H22B	0.0957	0.8826	0.7956	0.047*	
C23	0.2649 (7)	0.9579 (7)	0.8031 (4)	0.051 (2)	
H23A	0.3226	0.9837	0.8354	0.062*	
H23B	0.3024	0.9058	0.7705	0.062*	
C24	0.2266 (8)	1.0655 (7)	0.7835 (5)	0.056 (2)	
H24A	0.1927	1.1197	0.8167	0.067*	
H24B	0.1663	1.0404	0.7525	0.067*	
C25	0.3294 (10)	1.1297 (9)	0.7607 (6)	0.075 (3)	
H25A	0.3846	1.1634	0.7927	0.112*	
H25B	0.3012	1.1922	0.7445	0.112*	
H25C	0.3676	1.0744	0.7303	0.112*	
C26	0.6939 (6)	0.2858 (7)	0.7279 (3)	0.0408 (16)	
H26A	0.6140	0.3061	0.7219	0.049*	
H26B	0.7377	0.3579	0.7497	0.049*	
C27	0.6901 (9)	0.1948 (11)	0.7660 (4)	0.072 (3)	
H27A	0.7708	0.1779	0.7729	0.087*	0.4
H27B	0.6509	0.1216	0.7426	0.087*	0.4
H27C	0.7676	0.1668	0.7704	0.087*	0.6
H27D	0.6354	0.1270	0.7493	0.087*	0.6
C28A	0.630 (2)	0.224 (2)	0.8267 (8)	0.039 (6)	0.4
H28A	0.5561	0.2594	0.8246	0.047*	0.4
H28B	0.6172	0.1530	0.8421	0.047*	0.4
C29A	0.7227 (19)	0.312 (2)	0.8636 (10)	0.061 (6)	0.4
H29A	0.7973	0.2768	0.8612	0.091*	0.4
H29B	0.6991	0.3324	0.9042	0.091*	0.4
H29C	0.7300	0.3827	0.8487	0.091*	0.4
C28B	0.6464 (17)	0.268 (2)	0.8264 (7)	0.080 (9)	0.6
H28C	0.7048	0.3318	0.8441	0.096*	0.6
H28D	0.5733	0.3034	0.8206	0.096*	0.6
C29B	0.627 (2)	0.181 (3)	0.8667 (11)	0.172 (18)	0.6
H29D	0.5565	0.1306	0.8540	0.258*	0.6
H29E	0.6188	0.2251	0.9069	0.258*	0.6
H29F	0.6932	0.1328	0.8644	0.258*	0.6
C30	0.8787 (6)	0.2231 (6)	0.6741 (4)	0.0404 (16)	
H30A	0.8842	0.1626	0.6965	0.049*	
H30B	0.9086	0.1912	0.6350	0.049*	
C31	0.9577 (7)	0.3320 (9)	0.7044 (5)	0.069 (3)	
H31A	0.9315	0.3628	0.7444	0.082*	
H31B	0.9525	0.3940	0.6829	0.082*	
C32	1.0857 (7)	0.2986 (9)	0.7062 (5)	0.068 (3)	
H32A	1.1326	0.3662	0.7301	0.082*	
H32B	1.0886	0.2334	0.7257	0.082*	
C33	1.1404 (9)	0.2630 (11)	0.6472 (6)	0.093 (4)	
H33A	1.1033	0.1884	0.6255	0.139*	
H33B	1.2231	0.2546	0.6524	0.139*	
H33C	1.1303	0.3230	0.6256	0.139*	
C34	0.6902 (6)	0.1279 (6)	0.6335 (3)	0.0347 (14)	
H34A	0.7049	0.0675	0.6549	0.042*	
H34B	0.7279	0.1055	0.5954	0.042*	
C35	0.5576 (6)	0.1257 (6)	0.6223 (3)	0.0369 (14)	
H35A	0.5188	0.1534	0.6597	0.044*	
H35B	0.5417	0.1791	0.5971	0.044*	
C36	0.5090 (7)	0.0014 (7)	0.5931 (4)	0.0478 (18)	
H36A	0.5227	−0.0507	0.6192	0.057*	
H36B	0.5513	−0.0272	0.5569	0.057*	
C37	0.3771 (7)	−0.0056 (7)	0.5782 (4)	0.052 (2)	
H37A	0.3340	0.0151	0.6142	0.078*	
H37B	0.3521	−0.0852	0.5569	0.078*	
H37C	0.3622	0.0491	0.5540	0.078*	
C38	0.7337 (6)	0.3428 (6)	0.6346 (3)	0.0383 (15)	
H38A	0.7721	0.4167	0.6577	0.046*	
H38B	0.6502	0.3545	0.6321	0.046*	
C39	0.7830 (8)	0.3177 (8)	0.5717 (4)	0.059 (2)	
H39A	0.8636	0.2949	0.5728	0.071*	
H39B	0.7364	0.2517	0.5463	0.071*	
C40	0.7805 (8)	0.4279 (9)	0.5459 (5)	0.068 (3)	
H40A	0.7857	0.4021	0.5031	0.081*	
H40B	0.7046	0.4613	0.5543	0.081*	
C41	0.8687 (11)	0.5185 (12)	0.5664 (8)	0.111 (5)	
H41A	0.8663	0.5439	0.6088	0.166*	
H41B	0.8560	0.5847	0.5493	0.166*	
H41C	0.9443	0.4891	0.5550	0.166*	
C42	0.9468 (8)	0.0777 (8)	0.4729 (3)	0.053 (2)	
H42A	0.9359	0.1585	0.4697	0.063*	
H42B	0.8994	0.0234	0.4419	0.063*	
C43	0.9270 (8)	−0.0530 (7)	0.5351 (3)	0.050 (2)	
H43A	0.8791	−0.1102	0.5054	0.060*	
H43B	0.9027	−0.0618	0.5739	0.060*	

### Atomic displacement parameters (Å^2^)

**Table:**

	*U*^11^	*U*^22^	*U*^33^	*U*^12^	*U*^13^	*U*^23^
I1	0.0304 (2)	0.0365 (2)	0.0282 (2)	−0.01134 (16)	−0.00065 (15)	−0.00781 (16)
I2	0.0451 (2)	0.02683 (19)	0.01655 (16)	−0.00402 (16)	−0.00845 (14)	0.00788 (13)
I3	0.0544 (3)	0.0603 (3)	0.0397 (3)	−0.0281 (2)	0.0047 (2)	0.0250 (2)
Cu1	0.0394 (4)	0.0449 (5)	0.0183 (3)	−0.0246 (4)	−0.0077 (3)	0.0133 (3)
Cu2	0.0263 (3)	0.0242 (3)	0.0158 (3)	−0.0093 (3)	−0.0060 (2)	0.0080 (2)
Cu3	0.0208 (3)	0.0208 (3)	0.0159 (3)	−0.0045 (2)	−0.0005 (2)	0.0046 (2)
Cl1A	0.057 (2)	0.078 (3)	0.0226 (14)	−0.046 (2)	−0.0200 (14)	0.0287 (17)
Cl1B	0.057 (3)	0.058 (3)	0.022 (2)	−0.039 (3)	−0.020 (2)	0.021 (2)
Cl2	0.0199 (5)	0.0198 (5)	0.0127 (5)	−0.0122 (4)	−0.0068 (4)	0.0101 (4)
Cl3	0.0186 (5)	0.0145 (5)	0.0119 (5)	−0.0028 (4)	−0.0028 (4)	0.0022 (4)
Cl4	0.0096 (5)	0.0206 (5)	0.0122 (5)	−0.0027 (4)	0.0013 (4)	−0.0001 (4)
Cl5	0.0114 (4)	0.0122 (5)	0.0115 (4)	−0.0058 (4)	−0.0040 (3)	0.0075 (4)
O1	0.046 (3)	0.051 (3)	0.042 (3)	0.014 (2)	0.018 (2)	0.013 (2)
N1	0.029 (2)	0.034 (3)	0.018 (2)	−0.012 (2)	−0.0026 (18)	0.0065 (19)
N2	0.022 (2)	0.024 (2)	0.017 (2)	−0.0027 (17)	−0.0029 (16)	0.0074 (17)
N3	0.025 (2)	0.022 (2)	0.018 (2)	−0.0056 (18)	−0.0021 (17)	0.0081 (17)
N4	0.024 (2)	0.019 (2)	0.017 (2)	−0.0049 (17)	−0.0018 (16)	0.0041 (16)
N5	0.022 (2)	0.027 (2)	0.016 (2)	−0.0041 (18)	−0.0009 (16)	0.0036 (17)
N6	0.028 (2)	0.034 (3)	0.019 (2)	−0.010 (2)	−0.0032 (18)	0.0087 (19)
N7	0.020 (2)	0.039 (3)	0.043 (3)	0.002 (2)	0.003 (2)	0.010 (2)
N8	0.028 (3)	0.033 (3)	0.041 (3)	0.010 (2)	0.000 (2)	0.007 (2)
C1	0.022 (2)	0.020 (2)	0.023 (3)	−0.002 (2)	−0.004 (2)	0.005 (2)
C2	0.021 (2)	0.024 (3)	0.021 (2)	0.000 (2)	−0.0027 (19)	−0.002 (2)
C3	0.028 (3)	0.039 (3)	0.019 (3)	−0.013 (2)	−0.003 (2)	0.006 (2)
C4	0.027 (3)	0.022 (3)	0.020 (2)	−0.003 (2)	−0.009 (2)	0.008 (2)
C5	0.032 (3)	0.022 (3)	0.016 (2)	0.000 (2)	−0.005 (2)	0.005 (2)
C6	0.028 (3)	0.024 (3)	0.020 (2)	−0.002 (2)	−0.003 (2)	0.010 (2)
C7	0.033 (3)	0.036 (3)	0.022 (3)	−0.009 (2)	0.001 (2)	0.014 (2)
C8	0.036 (3)	0.038 (3)	0.022 (3)	−0.012 (3)	0.004 (2)	0.014 (2)
C9	0.024 (3)	0.030 (3)	0.023 (3)	−0.006 (2)	0.001 (2)	0.008 (2)
C10	0.026 (3)	0.091 (7)	0.059 (5)	−0.008 (4)	0.001 (3)	0.038 (5)
C11	0.046 (5)	0.138 (11)	0.168 (13)	−0.027 (6)	−0.026 (7)	0.125 (11)
C12A	0.039 (7)	0.059 (9)	0.052 (8)	−0.011 (6)	−0.011 (6)	0.036 (7)
C13A	0.081 (13)	0.119 (18)	0.18 (2)	−0.053 (13)	−0.055 (14)	0.13 (2)
C12B	0.08 (2)	0.08 (2)	0.054 (15)	−0.053 (18)	−0.005 (13)	0.006 (14)
C13B	0.12 (3)	0.077 (19)	0.057 (16)	0.021 (19)	0.001 (16)	0.002 (14)
C14	0.027 (3)	0.047 (4)	0.031 (3)	0.005 (3)	0.002 (2)	0.001 (3)
C15	0.046 (4)	0.074 (6)	0.039 (4)	0.014 (4)	0.007 (3)	−0.005 (4)
C16A	0.23 (4)	0.18 (4)	0.046 (14)	0.15 (3)	−0.06 (2)	−0.043 (18)
C17A	0.077 (15)	0.33 (5)	0.062 (13)	0.07 (2)	−0.019 (11)	−0.06 (2)
C16B	0.067 (16)	0.071 (18)	0.024 (11)	−0.005 (14)	−0.015 (10)	−0.008 (10)
C17B	0.071 (17)	0.12 (3)	0.053 (15)	0.003 (17)	0.008 (13)	0.001 (16)
C18	0.033 (3)	0.029 (3)	0.040 (3)	0.006 (3)	−0.004 (3)	0.008 (3)
C19	0.041 (4)	0.037 (4)	0.054 (4)	0.003 (3)	−0.019 (3)	0.010 (3)
C20	0.073 (6)	0.068 (6)	0.049 (5)	0.033 (5)	−0.029 (4)	−0.013 (4)
C21	0.081 (8)	0.139 (12)	0.051 (6)	0.033 (8)	0.012 (5)	0.026 (7)
C22	0.027 (3)	0.035 (3)	0.053 (4)	0.010 (3)	0.003 (3)	0.004 (3)
C23	0.034 (4)	0.033 (4)	0.086 (6)	0.005 (3)	0.003 (4)	0.008 (4)
C24	0.052 (5)	0.034 (4)	0.080 (6)	−0.002 (3)	−0.006 (4)	0.011 (4)
C25	0.071 (7)	0.046 (5)	0.109 (9)	−0.009 (5)	0.007 (6)	0.024 (5)
C26	0.034 (3)	0.050 (4)	0.036 (3)	0.016 (3)	−0.001 (3)	0.002 (3)
C27	0.058 (5)	0.125 (9)	0.048 (5)	0.058 (6)	0.011 (4)	0.035 (5)
C28A	0.047 (13)	0.032 (9)	0.031 (11)	−0.017 (8)	−0.004 (8)	−0.005 (8)
C29A	0.054 (13)	0.072 (15)	0.052 (12)	0.010 (11)	−0.003 (10)	0.003 (11)
C28B	0.025 (8)	0.18 (3)	0.054 (12)	0.036 (13)	0.009 (7)	0.048 (14)
C29B	0.14 (2)	0.34 (4)	0.121 (18)	0.19 (3)	0.102 (17)	0.17 (3)
C30	0.024 (3)	0.037 (4)	0.057 (4)	0.015 (3)	0.002 (3)	0.001 (3)
C31	0.032 (4)	0.061 (5)	0.092 (7)	0.013 (4)	−0.015 (4)	−0.024 (5)
C32	0.031 (4)	0.060 (6)	0.103 (8)	0.011 (4)	−0.010 (4)	−0.003 (5)
C33	0.040 (5)	0.080 (7)	0.125 (10)	−0.017 (5)	0.012 (6)	−0.037 (7)
C34	0.039 (3)	0.026 (3)	0.039 (3)	0.010 (3)	−0.002 (3)	0.006 (3)
C35	0.038 (3)	0.029 (3)	0.047 (4)	0.007 (3)	−0.001 (3)	0.013 (3)
C36	0.045 (4)	0.033 (4)	0.061 (5)	0.008 (3)	−0.003 (4)	0.002 (3)
C37	0.046 (4)	0.045 (4)	0.064 (5)	−0.004 (3)	−0.011 (4)	0.015 (4)
C38	0.032 (3)	0.025 (3)	0.060 (4)	0.011 (3)	0.004 (3)	0.013 (3)
C39	0.061 (5)	0.057 (5)	0.070 (6)	0.030 (4)	0.020 (4)	0.033 (5)
C40	0.049 (5)	0.064 (6)	0.106 (8)	0.017 (4)	0.008 (5)	0.047 (6)
C41	0.068 (8)	0.079 (8)	0.200 (18)	0.003 (6)	−0.021 (9)	0.063 (10)
C42	0.075 (6)	0.052 (5)	0.038 (4)	0.026 (4)	0.007 (4)	0.019 (3)
C43	0.066 (5)	0.045 (4)	0.039 (4)	−0.013 (4)	0.019 (4)	0.014 (3)

### Geometric parameters (Å, º)

**Table:**

I1—C2	2.063 (5)	C17B—H17E	0.9600
I2—C5	2.069 (5)	C17B—H17F	0.9600
I3—C8	2.060 (6)	C18—C19	1.523 (9)
Cu1—N1	1.926 (5)	C18—H18A	0.9700
Cu1—N6	1.926 (5)	C18—H18B	0.9700
Cu1—Cl1A	2.289 (3)	C19—C20	1.550 (13)
Cu1—Cl1B	2.335 (5)	C19—H19A	0.9700
Cu1—Cl4	2.6258 (14)	C19—H19B	0.9700
Cu1—Cl5	2.6478 (14)	C20—C21	1.555 (17)
Cu2—N2	1.933 (4)	C20—H20A	0.9700
Cu2—N3	1.936 (4)	C20—H20B	0.9700
Cu2—Cl2	2.3458 (12)	C21—H21A	0.9600
Cu2—Cl5	2.5808 (12)	C21—H21B	0.9600
Cu2—Cl4	2.6306 (13)	C21—H21C	0.9600
Cu3—N4	1.945 (4)	C22—C23	1.524 (10)
Cu3—N5	1.951 (5)	C22—H22A	0.9700
Cu3—Cl3	2.3337 (12)	C22—H22B	0.9700
Cu3—Cl4	2.5016 (13)	C23—C24	1.510 (11)
Cu3—Cl5	2.7607 (12)	C23—H23A	0.9700
O1—C43	1.412 (10)	C23—H23B	0.9700
O1—C42	1.433 (9)	C24—C25	1.510 (13)
N1—N2	1.347 (6)	C24—H24A	0.9700
N1—C3	1.353 (7)	C24—H24B	0.9700
N2—C1	1.356 (7)	C25—H25A	0.9600
N3—C6	1.337 (7)	C25—H25B	0.9600
N3—N4	1.364 (6)	C25—H25C	0.9600
N4—C4	1.339 (7)	C26—C27	1.526 (12)
N5—C9	1.333 (7)	C26—H26A	0.9700
N5—N6	1.369 (6)	C26—H26B	0.9700
N6—C7	1.344 (7)	C27—C28A	1.542 (17)
N7—C22	1.516 (9)	C27—C28B	1.560 (16)
N7—C18	1.518 (9)	C27—H27A	0.9700
N7—C10	1.519 (9)	C27—H27B	0.9700
N7—C14	1.521 (8)	C27—H27C	0.9700
N8—C34	1.517 (8)	C27—H27D	0.9700
N8—C26	1.517 (9)	C28A—C29A	1.522 (17)
N8—C38	1.523 (9)	C28A—H28A	0.9600
N8—C30	1.523 (8)	C28A—H28B	0.9700
C1—C2	1.394 (7)	C29A—H29A	0.9600
C1—H1	0.9300	C29A—H29B	0.9600
C2—C3	1.382 (8)	C29A—H29C	0.9600
C3—H3	0.9300	C28B—C29B	1.538 (18)
C4—C5	1.387 (8)	C28B—H28C	0.9700
C4—H4	0.9300	C28B—H28D	0.9700
C5—C6	1.381 (7)	C29B—H29D	0.9600
C6—H6	0.9300	C29B—H29E	0.9600
C7—C8	1.389 (8)	C29B—H29F	0.9600
C7—H7	0.9300	C30—C31	1.521 (11)
C8—C9	1.401 (8)	C30—H30A	0.9700
C9—H9	0.9300	C30—H30B	0.9700
C10—C11	1.506 (14)	C31—C32	1.531 (11)
C10—H10A	0.9700	C31—H31A	0.9700
C10—H10B	0.9700	C31—H31B	0.9700
C11—C12B	1.514 (16)	C32—C33	1.486 (16)
C11—C12A	1.567 (14)	C32—H32A	0.9700
C11—H11A	0.9700	C32—H32B	0.9700
C11—H11B	0.9700	C33—H33A	0.9600
C11—H11C	0.9700	C33—H33B	0.9600
C11—H11D	0.9700	C33—H33C	0.9600
C12A—C13A	1.592 (15)	C34—C35	1.525 (9)
C12A—H12A	0.9700	C34—H34A	0.9700
C12A—H12B	0.9700	C34—H34B	0.9700
C13A—H13A	0.9600	C35—C36	1.504 (10)
C13A—H13B	0.9600	C35—H35A	0.9700
C13A—H13C	0.9600	C35—H35B	0.9700
C12B—C13B	1.533 (18)	C36—C37	1.529 (11)
C12B—H12C	0.9700	C36—H36A	0.9700
C12B—H12D	0.9600	C36—H36B	0.9700
C13B—H13D	0.9600	C37—H37A	0.9600
C13B—H13E	0.9600	C37—H37B	0.9600
C13B—H13F	0.9600	C37—H37C	0.9600
C14—C15	1.522 (10)	C38—C39	1.538 (11)
C14—H14A	0.9700	C38—H38A	0.9700
C14—H14B	0.9700	C38—H38B	0.9700
C15—C16A	1.548 (17)	C39—C40	1.534 (12)
C15—C16B	1.561 (18)	C39—H39A	0.9700
C15—H15A	0.9700	C39—H39B	0.9700
C15—H15B	0.9700	C40—C41	1.397 (16)
C15—H15C	0.9700	C40—H40A	0.9700
C15—H15D	0.9700	C40—H40B	0.9700
C16A—C17A	1.517 (18)	C41—H41A	0.9600
C16A—H16A	0.9700	C41—H41B	0.9600
C16A—H16B	0.9700	C41—H41C	0.9600
C17A—H17A	0.9600	C42—C43^i^	1.484 (12)
C17A—H17B	0.9600	C42—H42A	0.9700
C17A—H17C	0.9600	C42—H42B	0.9700
C16B—C17B	1.548 (19)	C43—C42^i^	1.484 (12)
C16B—H16C	0.9600	C43—H43A	0.9700
C16B—H16D	0.9700	C43—H43B	0.9700
C17B—H17D	0.9600		
			
N1—Cu1—N6	173.7 (2)	H17D—C17B—H17E	109.5
N1—Cu1—Cl1A	90.85 (16)	C16B—C17B—H17F	109.5
N6—Cu1—Cl1A	92.47 (16)	H17D—C17B—H17F	109.5
N1—Cu1—Cl1B	93.60 (18)	H17E—C17B—H17F	109.5
N6—Cu1—Cl1B	91.57 (18)	N7—C18—C19	114.6 (6)
N1—Cu1—Cl4	86.25 (15)	N7—C18—H18A	108.6
N6—Cu1—Cl4	87.74 (15)	C19—C18—H18A	108.6
Cl1A—Cu1—Cl4	140.10 (15)	N7—C18—H18B	108.6
Cl1B—Cu1—Cl4	163.21 (19)	C19—C18—H18B	108.6
N1—Cu1—Cl5	90.42 (15)	H18A—C18—H18B	107.6
N6—Cu1—Cl5	90.84 (16)	C18—C19—C20	109.7 (6)
Cl1A—Cu1—Cl5	136.96 (15)	C18—C19—H19A	109.7
Cl1B—Cu1—Cl5	113.90 (19)	C20—C19—H19A	109.7
Cl4—Cu1—Cl5	82.89 (4)	C18—C19—H19B	109.7
N2—Cu2—N3	170.5 (2)	C20—C19—H19B	109.7
N2—Cu2—Cl2	92.12 (14)	H19A—C19—H19B	108.2
N3—Cu2—Cl2	93.63 (13)	C19—C20—C21	113.1 (8)
N2—Cu2—Cl5	92.75 (14)	C19—C20—H20A	109.0
N3—Cu2—Cl5	90.56 (14)	C21—C20—H20A	109.0
Cl2—Cu2—Cl5	122.39 (5)	C19—C20—H20B	109.0
N2—Cu2—Cl4	85.89 (14)	C21—C20—H20B	109.0
N3—Cu2—Cl4	85.60 (14)	H20A—C20—H20B	107.8
Cl2—Cu2—Cl4	153.50 (5)	C20—C21—H21A	109.5
Cl5—Cu2—Cl4	84.10 (4)	C20—C21—H21B	109.5
N4—Cu3—N5	176.18 (19)	H21A—C21—H21B	109.5
N4—Cu3—Cl3	93.20 (13)	C20—C21—H21C	109.5
N5—Cu3—Cl3	90.59 (14)	H21A—C21—H21C	109.5
N4—Cu3—Cl4	86.82 (14)	H21B—C21—H21C	109.5
N5—Cu3—Cl4	90.04 (14)	N7—C22—C23	115.8 (5)
Cl3—Cu3—Cl4	151.28 (5)	N7—C22—H22A	108.3
N4—Cu3—Cl5	88.00 (14)	C23—C22—H22A	108.3
N5—Cu3—Cl5	89.45 (14)	N7—C22—H22B	108.3
Cl3—Cu3—Cl5	125.75 (5)	C23—C22—H22B	108.3
Cl4—Cu3—Cl5	82.96 (4)	H22A—C22—H22B	107.4
Cu3—Cl4—Cu1	82.74 (4)	C24—C23—C22	111.7 (6)
Cu3—Cl4—Cu2	83.50 (4)	C24—C23—H23A	109.3
Cu1—Cl4—Cu2	79.97 (4)	C22—C23—H23A	109.3
Cu2—Cl5—Cu1	80.47 (4)	C24—C23—H23B	109.3
Cu2—Cl5—Cu3	79.51 (3)	C22—C23—H23B	109.3
Cu1—Cl5—Cu3	77.60 (4)	H23A—C23—H23B	107.9
C43—O1—C42	109.8 (6)	C23—C24—C25	111.2 (8)
N2—N1—C3	108.4 (5)	C23—C24—H24A	109.4
N2—N1—Cu1	122.7 (4)	C25—C24—H24A	109.4
C3—N1—Cu1	128.8 (4)	C23—C24—H24B	109.4
N1—N2—C1	108.5 (4)	C25—C24—H24B	109.4
N1—N2—Cu2	120.7 (3)	H24A—C24—H24B	108.0
C1—N2—Cu2	130.7 (4)	C24—C25—H25A	109.5
C6—N3—N4	108.1 (4)	C24—C25—H25B	109.5
C6—N3—Cu2	129.9 (4)	H25A—C25—H25B	109.5
N4—N3—Cu2	122.0 (3)	C24—C25—H25C	109.5
C4—N4—N3	108.2 (4)	H25A—C25—H25C	109.5
C4—N4—Cu3	129.9 (4)	H25B—C25—H25C	109.5
N3—N4—Cu3	121.9 (3)	N8—C26—C27	115.2 (6)
C9—N5—N6	108.6 (4)	N8—C26—H26A	108.5
C9—N5—Cu3	131.5 (4)	C27—C26—H26A	108.5
N6—N5—Cu3	119.9 (3)	N8—C26—H26B	108.5
C7—N6—N5	108.1 (5)	C27—C26—H26B	108.5
C7—N6—Cu1	128.9 (4)	H26A—C26—H26B	107.5
N5—N6—Cu1	122.9 (4)	C26—C27—C28A	119.7 (14)
C22—N7—C18	110.2 (5)	C26—C27—C28B	102.4 (11)
C22—N7—C10	107.2 (5)	C26—C27—H27A	107.4
C18—N7—C10	111.3 (6)	C28A—C27—H27A	107.4
C22—N7—C14	110.6 (5)	C26—C27—H27B	107.4
C18—N7—C14	106.9 (5)	C28A—C27—H27B	107.4
C10—N7—C14	110.7 (5)	H27A—C27—H27B	106.9
C34—N8—C26	110.9 (5)	C26—C27—H27C	111.3
C34—N8—C38	110.5 (5)	C28B—C27—H27C	111.3
C26—N8—C38	107.2 (5)	C26—C27—H27D	111.3
C34—N8—C30	105.6 (5)	C28B—C27—H27D	111.3
C26—N8—C30	111.2 (5)	H27C—C27—H27D	109.2
C38—N8—C30	111.5 (5)	C29A—C28A—C27	100.9 (15)
N2—C1—C2	108.6 (5)	C29A—C28A—H28A	111.6
N2—C1—H1	125.7	C27—C28A—H28A	111.6
C2—C1—H1	125.7	C29A—C28A—H28B	111.6
C3—C2—C1	105.2 (5)	C27—C28A—H28B	111.6
C3—C2—I1	124.5 (4)	H28A—C28A—H28B	109.4
C1—C2—I1	129.8 (4)	C28A—C29A—H29A	109.5
N1—C3—C2	109.2 (5)	C28A—C29A—H29B	109.5
N1—C3—H3	125.4	H29A—C29A—H29B	109.5
C2—C3—H3	125.4	C28A—C29A—H29C	109.5
N4—C4—C5	109.2 (5)	H29A—C29A—H29C	109.5
N4—C4—H4	125.4	H29B—C29A—H29C	109.5
C5—C4—H4	125.4	C29B—C28B—C27	106.9 (15)
C6—C5—C4	105.0 (5)	C29B—C28B—H28C	110.3
C6—C5—I2	127.8 (4)	C27—C28B—H28C	110.4
C4—C5—I2	127.0 (4)	C29B—C28B—H28D	110.4
N3—C6—C5	109.6 (5)	C27—C28B—H28D	110.4
N3—C6—H6	125.2	H28C—C28B—H28D	108.6
C5—C6—H6	125.2	C28B—C29B—H29D	109.5
N6—C7—C8	109.2 (5)	C28B—C29B—H29E	109.5
N6—C7—H7	125.4	H29D—C29B—H29E	109.5
C8—C7—H7	125.4	C28B—C29B—H29F	109.5
C7—C8—C9	105.0 (5)	H29D—C29B—H29F	109.5
C7—C8—I3	124.3 (4)	H29E—C29B—H29F	109.5
C9—C8—I3	130.7 (4)	C31—C30—N8	115.1 (6)
N5—C9—C8	109.1 (5)	C31—C30—H30A	108.5
N5—C9—H9	125.5	N8—C30—H30A	108.5
C8—C9—H9	125.5	C31—C30—H30B	108.5
C11—C10—N7	115.4 (7)	N8—C30—H30B	108.5
C11—C10—H10A	108.4	H30A—C30—H30B	107.5
N7—C10—H10A	108.4	C30—C31—C32	110.0 (7)
C11—C10—H10B	108.4	C30—C31—H31A	109.7
N7—C10—H10B	108.4	C32—C31—H31A	109.7
H10A—C10—H10B	107.5	C30—C31—H31B	109.7
C10—C11—C12B	132.7 (18)	C32—C31—H31B	109.7
C10—C11—C12A	103.6 (10)	H31A—C31—H31B	108.2
C10—C11—H11A	111.0	C33—C32—C31	114.8 (10)
C12A—C11—H11A	111.0	C33—C32—H32A	108.6
C10—C11—H11B	111.0	C31—C32—H32A	108.6
C12A—C11—H11B	111.0	C33—C32—H32B	108.6
H11A—C11—H11B	109.0	C31—C32—H32B	108.6
C10—C11—H11C	104.1	H32A—C32—H32B	107.6
C12B—C11—H11C	104.1	C32—C33—H33A	109.5
C10—C11—H11D	104.1	C32—C33—H33B	109.5
C12B—C11—H11D	104.1	H33A—C33—H33B	109.5
H11C—C11—H11D	105.5	C32—C33—H33C	109.5
C11—C12A—C13A	103.6 (12)	H33A—C33—H33C	109.5
C11—C12A—H12A	111.1	H33B—C33—H33C	109.5
C13A—C12A—H12A	111.1	N8—C34—C35	116.1 (5)
C11—C12A—H12B	111.0	N8—C34—H34A	108.3
C13A—C12A—H12B	111.0	C35—C34—H34A	108.3
H12A—C12A—H12B	109.0	N8—C34—H34B	108.3
C12A—C13A—H13A	109.5	C35—C34—H34B	108.3
C12A—C13A—H13B	109.5	H34A—C34—H34B	107.4
H13A—C13A—H13B	109.5	C36—C35—C34	110.5 (6)
C12A—C13A—H13C	109.5	C36—C35—H35A	109.5
H13A—C13A—H13C	109.5	C34—C35—H35A	109.5
H13B—C13A—H13C	109.5	C36—C35—H35B	109.5
C11—C12B—C13B	102.0 (19)	C34—C35—H35B	109.5
C11—C12B—H12C	111.4	H35A—C35—H35B	108.1
C13B—C12B—H12C	111.4	C35—C36—C37	113.0 (6)
C11—C12B—H12D	111.4	C35—C36—H36A	109.0
C13B—C12B—H12D	111.4	C37—C36—H36A	109.0
H12C—C12B—H12D	109.2	C35—C36—H36B	109.0
C12B—C13B—H13D	109.5	C37—C36—H36B	109.0
C12B—C13B—H13E	109.5	H36A—C36—H36B	107.8
H13D—C13B—H13E	109.5	C36—C37—H37A	109.5
C12B—C13B—H13F	109.5	C36—C37—H37B	109.5
H13D—C13B—H13F	109.5	H37A—C37—H37B	109.5
H13E—C13B—H13F	109.5	C36—C37—H37C	109.5
N7—C14—C15	116.5 (6)	H37A—C37—H37C	109.5
N7—C14—H14A	108.2	H37B—C37—H37C	109.5
C15—C14—H14A	108.2	N8—C38—C39	115.2 (5)
N7—C14—H14B	108.2	N8—C38—H38A	108.5
C15—C14—H14B	108.2	C39—C38—H38A	108.5
H14A—C14—H14B	107.3	N8—C38—H38B	108.5
C14—C15—C16A	108.5 (11)	C39—C38—H38B	108.5
C14—C15—C16B	109.2 (15)	H38A—C38—H38B	107.5
C14—C15—H15A	110.0	C40—C39—C38	111.0 (7)
C16A—C15—H15A	110.0	C40—C39—H39A	109.4
C14—C15—H15B	110.0	C38—C39—H39A	109.4
C16A—C15—H15B	110.0	C40—C39—H39B	109.4
H15A—C15—H15B	108.4	C38—C39—H39B	109.4
C14—C15—H15C	109.8	H39A—C39—H39B	108.0
C16B—C15—H15C	109.8	C41—C40—C39	116.4 (10)
C14—C15—H15D	109.8	C41—C40—H40A	108.2
C16B—C15—H15D	109.8	C39—C40—H40A	108.2
H15C—C15—H15D	108.3	C41—C40—H40B	108.2
C17A—C16A—C15	115.9 (18)	C39—C40—H40B	108.2
C17A—C16A—H16A	108.3	H40A—C40—H40B	107.3
C15—C16A—H16A	108.3	C40—C41—H41A	109.5
C17A—C16A—H16B	108.3	C40—C41—H41B	109.5
C15—C16A—H16B	108.3	H41A—C41—H41B	109.5
H16A—C16A—H16B	107.4	C40—C41—H41C	109.5
C16A—C17A—H17A	109.5	H41A—C41—H41C	109.5
C16A—C17A—H17B	109.5	H41B—C41—H41C	109.5
H17A—C17A—H17B	109.5	O1—C42—C43^i^	110.4 (6)
C16A—C17A—H17C	109.5	O1—C42—H42A	109.6
H17A—C17A—H17C	109.5	C43^i^—C42—H42A	109.6
H17B—C17A—H17C	109.5	O1—C42—H42B	109.6
C17B—C16B—C15	106.0 (19)	C43^i^—C42—H42B	109.6
C17B—C16B—H16C	110.5	H42A—C42—H42B	108.1
C15—C16B—H16C	110.5	O1—C43—C42^i^	110.5 (6)
C17B—C16B—H16D	110.5	O1—C43—H43A	109.6
C15—C16B—H16D	110.5	C42^i^—C43—H43A	109.6
H16C—C16B—H16D	108.7	O1—C43—H43B	109.6
C16B—C17B—H17D	109.5	C42^i^—C43—H43B	109.6
C16B—C17B—H17E	109.5	H43A—C43—H43B	108.1

Symmetry code: (i) −*x*+2, −*y*, −*z*+1.
